# Epidemic preparedness and innovations in digital healthcare: enhancing post-pandemic speech-language pathology services for child and adolescent mental health in Qatar

**DOI:** 10.1186/s12913-024-10989-y

**Published:** 2024-05-28

**Authors:** Abdullah Amro, Hanan Abdallah Kotkot, Yahia Albobali, Prem Chandra, Yasser Saeed Khan

**Affiliations:** 1 Child and Adolescent Mental Health Service, Hamad Medical Corporation, Doha, Qatar; 2 Medical Research Centre, Hamad Medical Corporation, Doha, Qatar; 3 College of Medicine, Qatar University, Doha, Qatar

**Keywords:** Mental health, Speech-Language pathology, Epidemic, Telehealth, Digital health, Children, Adolescents, Pandemic

## Abstract

**Background:**

This paper discusses the critical importance of epidemic preparedness and innovations in digital health care by examining the transformative impact on speech-language pathology (SLP) services in a specialist outpatient child and adolescent service (CAMHS).

**Method:**

This retrospective review analyzes referral data from three periods: pre-pandemic (15 March 2019–14 March 2020), pandemic (15 March 2020–14 March 2021), and post-pandemic (15 March 2021–14 March 2022). Statistical analyses assess trends in referrals and diagnoses during these periods. Feedback was also obtained from Parents of children who received virtual consultations during the pandemic.

**Results:**

The results reveal an increase in the demand for SLP services during and after the pandemic, with a surge in referrals (increased from 9.7 to 12.9% when compared pre-pandemic to post-pandemic periods; Chi-Square value 3.33, *P* = 0.068) for children with social communication challenges and autism spectrum disorder. Phone and video consultations were effectively adopted. Feedback from families shows a positive response (69%–98% of participants responded as strongly agreed and agreed on various items listed in feedback form specifically designed in line with the service objectives) to telehealth interventions, with many parents finding virtual consultations effective and helpful.

**Conclusions:**

The study emphasizes the importance of telehealth SLP services in meeting the increasing demand for mental health interventions among children and adolescents. It suggests integrating telehealth into clinical practice beyond the pandemic and highlights the need for long-term evaluation and addressing potential barriers to access.

**Supplementary Information:**

The online version contains supplementary material available at 10.1186/s12913-024-10989-y.

## Introduction

Epidemic preparedness regarding children’s development and mental health has gained unprecedented importance, particularly in the wake of the COVID-19 pandemic. The pandemic rendered children and adolescents with neurodevelopmental and mental disorders vulnerable in the sense that they faced unique challenges, disrupting their routines, isolating them from peers, and affecting their access to essential healthcare services. With nearly half of all mental disorders beginning by the age of 14 years [1], and up to 20% of children and adolescents who have a disabling mental disorder each year globally [2] the vulnerability of this specific patient population to epidemic disruptions is undeniable. Mental health services for children and adolescents around the world have been under strain for several years, more so since the onset of the COVID-19 pandemic [3]. There has been an escalation in the treatment demand for mental health problems in childhood and adolescence [4–6]. A study in Ireland reported a significant increase in the number of referrals to a child and adolescent mental health service one year after the COVID-19 pandemic [7].

The COVID-19 pandemic and the associated social restrictions rendered children and adolescents with neurodevelopmental disorders particularly vulnerable [8, 9]. There have been reports of an increase in externalizing behaviors in children with neurodevelopmental disorders due to reduced therapy [10]. Given the higher prevalence of communication challenges in this group of individuals, the need and delivery of speech-language pathology (SLP) services gained paramount importance. It is understood that precautionary measures taken during the pandemic (wearing masks, canceling in-person activities, social isolation) may have impacted children’s communication and language skills during their critical development years [11].

In this paper, we report the impact of the COVID-19 pandemic on the SLP referral trends in a specialist outpatient child and adolescent service (CAMHS) in Hamad Medical Corporation (HMC), Qatar. HMC is the largest public healthcare provider in Qatar, and its multidisciplinary CAMHS service receives referrals from multiple sources including primary care, schools, and general hospitals [12]. We also describe how the SLP service in HMC CAMHS remained functional during the COVID-19 pandemic through the utilisation of virtual modalities of care. It outlines how the demand for speech-language interventions for children and adolescents with neurodevelopmental and mental disorders surged during the pandemic, underscoring the critical need for innovative approaches such as telehealth to bridge the service gap.

By examining the impact of the pandemic on SLP referral trends and evaluating the efficacy of telehealth interventions, this study contributes to the ongoing discourse on advancing epidemic preparedness for health systems, especially concerning the vulnerable population of children and adolescents. As the world navigates post-pandemic challenges, it is imperative to prioritize the developmental and mental well-being of young individuals through proactive and adaptable approaches within healthcare systems. The feedback provided by families who received care during the pandemic and future directions are also discussed.

## Method

### Ethical considerations

This service evaluation initiative involved a retrospective analysis of existing data. No new patients were recruited. The data used was fully anonymized and not linked to any patient-identifiable information to ensure data protection and confidentiality. The study did not meet the requirements for ethical approval by the institutional review board and necessary departmental approval was obtained from the leadership of mental health services, in line with the advice provided by the medical research centre.

### Referral data

The referral pathway to SLP services in HMC CAMHS is such that most referrals to the two speech-language pathologists are made internally by other members of the multidisciplinary team. A typical example could be a child or young person receiving a diagnosis of autism spectrum disorder (ASD) by the ASD assessment team and then being referred to SLP services for follow-on work focusing on communication. Occasionally, referrals may be received from external providers for children and young people who may have been discharged from other services e.g., Developmental Paediatrics due to age limit, but may require continued SLP intervention. In this case, the referral is discussed by the CAMHS Triage team with the speech-language pathologists in the team to ensure it is appropriate for the service.

We compared three different time periods to ascertain if any trends existed in the number and nature of referrals to SLP services. COVID-19 restrictions were implemented in the state of Qatar during the second week of March 2020. The first period reviewed was 15 March 2019–14 March 2020 (pre-pandemic period), followed by 15th March 2020–14th March 2021 (pandemic period) and 15th March 2021- 14th March 2022 (post-pandemic period).

### Statistical analyses

Anonymous data were collected using a specific standard data collection tool and entered into a database designed in view of the study design and objectives. Descriptive statistics were used to summarize and determine the participants’ characteristics and distribution of various considered parameters related to demographics and diagnosis of the participants across three periods (Pre-pandemic period, Pandemic period, and Post-pandemic period). Quantitative data were presented using mean and standard deviation (SD). Categorical data were summarized using frequencies and percentages. The Mantel-Haenszel Chi-Square test was employed to examine and statistically evaluate the linear trend in the percentage (%) of referrals to SLP. Associations between two or more qualitative variables across three periods were examined and assessed using Pearson Chi-square or Yates corrected Chi-Square tests as appropriate. Quantitative data (mean age) measured across three periods were compared using one-way analysis of variance (ANOVA) followed by the Bonferroni multiple-comparison test. The most crucial findings were graphically represented using appropriate statistical graphs. All P values presented were two-tailed, and P values < 0.05 was considered statistically significant. All Statistical analyses were done using statistical packages SPSS version 27.0 (Armonk, NY: IBM Corp) and Epi Info 2000 (Centers for Disease Control and Prevention, Atlanta, GA).

### Feedback questionnaires

We collected responses from parents on feedback questionnaires designed to obtain their views about the quality of the service received. The original questionnaire was developed in English Language which was forward-translated into Arabic and further back-translated into English by experienced Psychiatrists in the local Child and Adolescent mental health services with excellent proficiency in both English and Arabic languages. It was ensured that the original and the translated versions achieved conceptual and linguistic equivalence. The translated questionnaires were piloted on the first five respondents. Their understanding of each item on the questionnaire and its response was explored to ensure the translated items retained the same meaning as the original items. The questionnaires comprised six questions and were offered in English or Arabic language.

## Results

### Referral and diagnosis trends

**Fig. 1:**
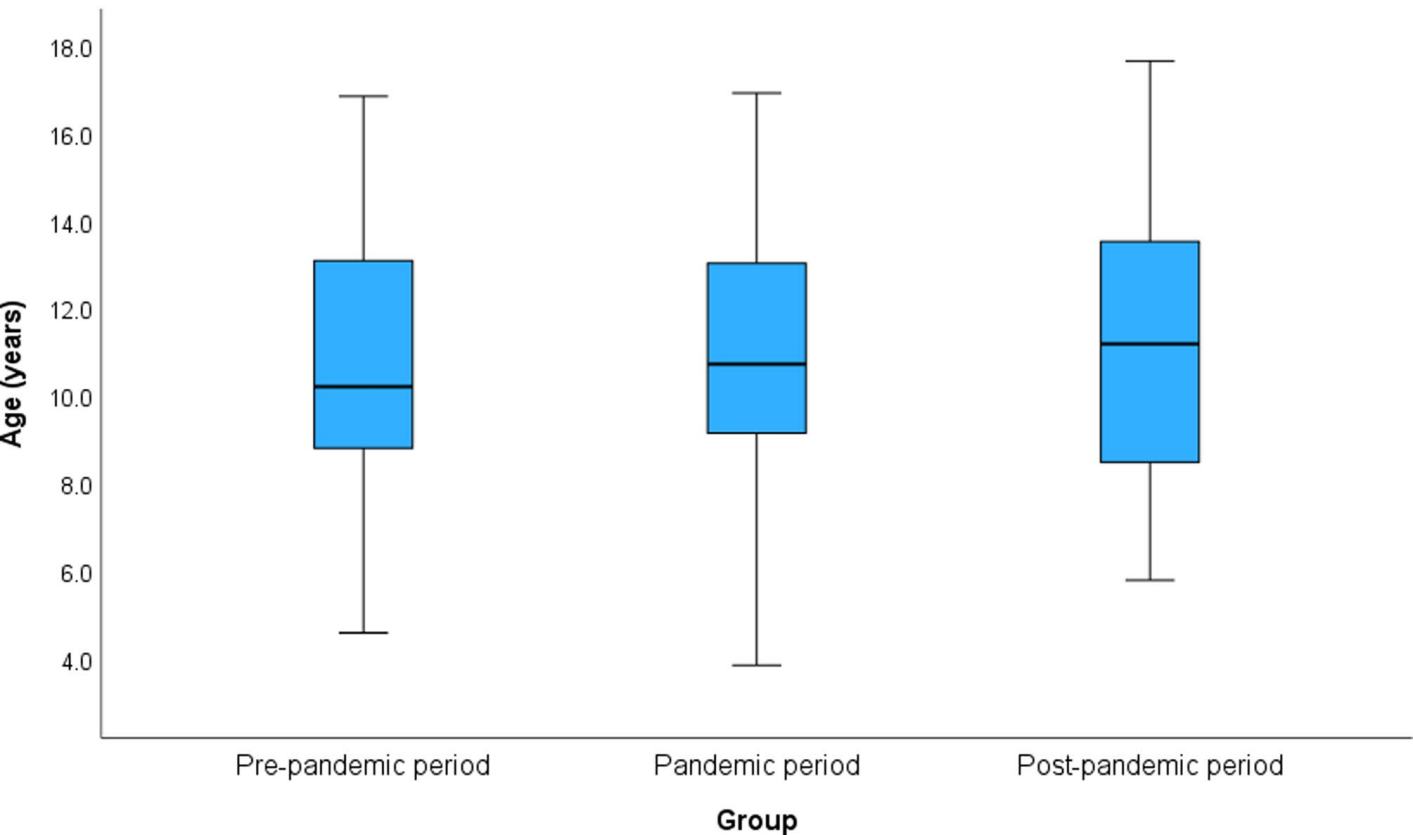
Box plot depicts Age distribution across the c periods

Two hundred forty-eight children and young people were referred to the SLP service in HMC CAMHS over the 3 years. Of these 248, 175 were boys and 73 were girls. The age range was 3–18 years. Figure 1 displays a box plot illustrating the distribution of ages across the three periods. Children and young people referred to SLP services were categorized into three groups based on age range: young children (Group 1: aged 3 years to < 9 years), older children (Group 2: aged 9 years to < 13 years), and adolescents (Group 3: aged 13 years to < 18 years).

Expatriates formed 70.6% and natives 29.4% of the total patients, and the percentage of patients was observed to be significantly (*P* = 0.008) lower in the pandemic period (58%) compared to the pre-pandemic period (82%) and post-pandemic period (73%) (Table 1).

**Table 1:** Demographics and primary diagnosis

		Pre-pandemic period	Pandemic period	Post-pandemic period	*P*-value
Number of patients		61 (24.6%)	77 (31.0%)	110 (44.4%)	
**Age (years)**, mean ± SD		10.87 ± 2.76	11.03 ± 2.86	11.10 ± 2.92	0.876^†^
Age Group	3 to < 9 years	16 (26.2%)	18 (23.4%)	30 (27.3%)	0.738^*^
	9 to < 13 years	29 (47.6%)	39 (50.6%)	45 (40.9%)	
	13 to 18 years	16 (26.2%)	20 (26.0%)	35 (31.8%)	
**Nationality**	Native	11 (18%)	32 (42%)	30 (27%)	0.008^*^
	Expat	50 (82%)	45 (58%)	80 (73%)	
**Gender**	Male	48 (79%)	54 (70%)	73 (66%)	0.237^*^
	Female	13 (21%)	23 (30%)	37 (34%)	
***Primary diagnosis***					
Autism Spectrum Disorder		32 (53%)	28 (36%)	51 (47%)	0.088^¥^
ADHD		14 (23%)	28 (36%)	22 (20%)	
Social (Pragmatic) Communication Disorder		8 (13%)	13 (17%)	28 (25%)	
Others		2 (3%)	6 (8%)	8 (7%)	
Intellectual/ Learning disability		5 (8%)	2 (3%)	1 (1%)	

^*†*^*ANOVA test*, ^*^Pearson Chi-Square test, ^*¥*^*Yates corrected Chi-Square test*

**Fig. 2:**
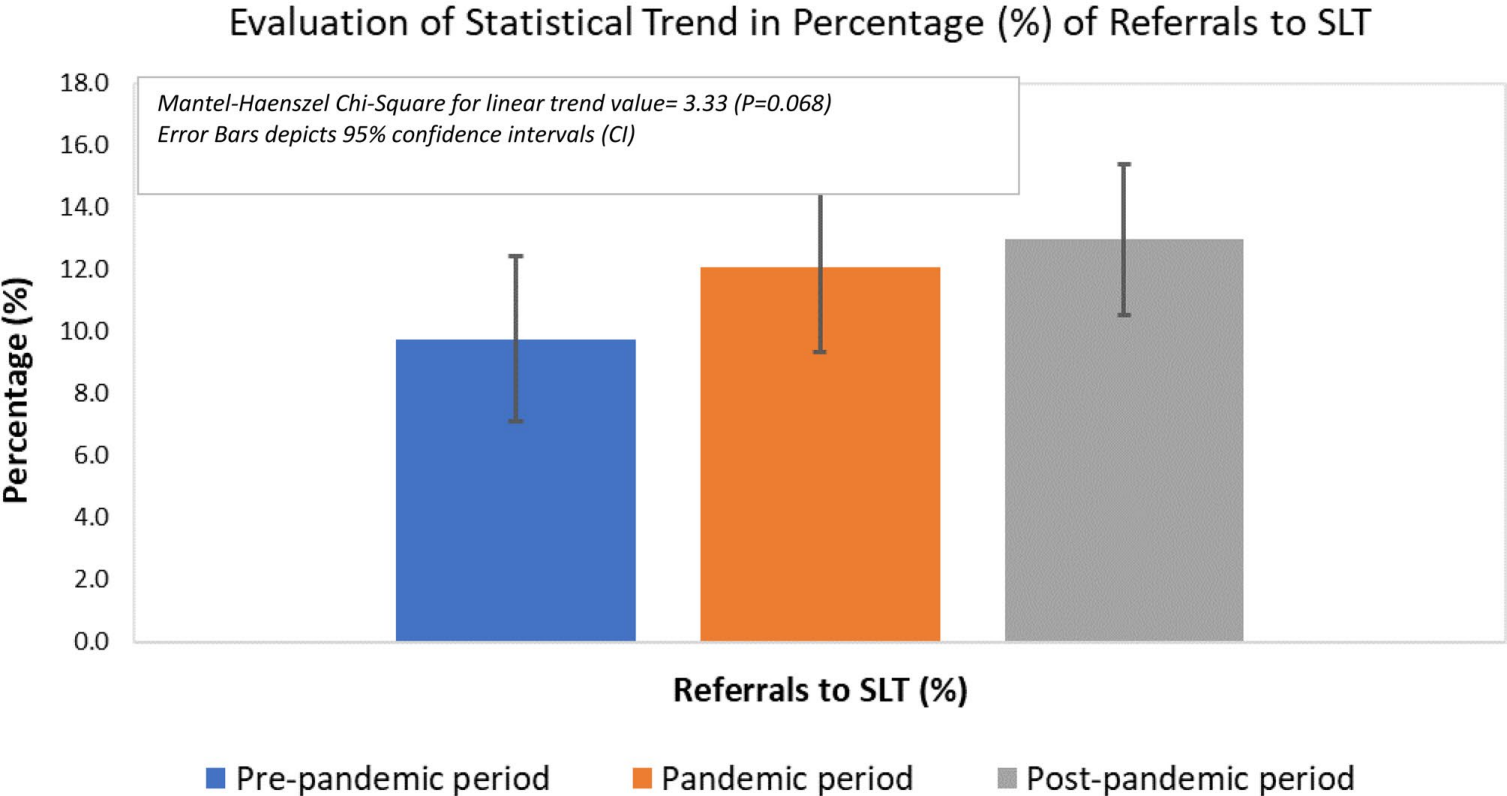
Evaluation of statistical trend in percentage (%) of referrals to SLT

Mantel-Haenszel Chi-Square test indicated an increased linear trend in the percentage of referrals to SLP (pre-pandemic 9.7%, pandemic 12.1% and post-pandemic, 13%); however, this difference was statistically insignificant (Chi-Square for linear trend 3.33, *P* = 0.068) as shown in Table 2; Fig. 2. Moreover, a consistent increase was noted over the 3 periods in the absolute percentage of patients referred to the SLP service. It increased by 26.2% between the pre-pandemic and pandemic periods and a further increase of 42.8% was noted between the pandemic and the post-pandemic periods. Furthermore, the number escalated by 80.3% when the post and pre-pandemic periods were compared (Table 1).

**Table 2:** Statistical assessment of linear trend in percentage (%) of referrals to SLT using Mantel-Haenszel Chi-Square test

	Accepted referrals	Referrals to SLT	Referrals to SLT (%)	95% CI for Referrals to SLT (%)	Mantel-Haenszel Chi-Square for linear trend	*P*-value
**Pre-pandemic period**	625	61	9.76	7.67, 12.34		
**Pandemic period**	636	77	12.11	9.80, 14.87	3.33	0.068
**Post-pandemic period**	847	110	12.99	10.90, 15.42		

CI: Confidence interval

A breakdown of diagnoses for the 248 children and young people was also included in Table 1. In the case of coexisting conditions, the primary diagnosis with the most impairing impact on the functioning of the child/young person was prioritised. Social (Pragmatic) communication disorder is mentioned separately given its relevance with CAMHS. Stuttering, Language Disorder, and Speech Sound Disorder were included in the “others” category. The majority of children and young people referred over the 3 years had a primary diagnosis of autism spectrum disorder followed by Attention deficit and hyperactivity disorder (ADHD), social (pragmatic) communication disorder, others, and intellectual/learning disability in decreasing order. The largest and most consistent increase over the three years was noted in the referrals received for patients with social (pragmatic) communication disorders. Various specific primary diagnoses outlined showed no significant association across three periods (*P* = 0.088).

### Clinical activity and contact modalities during the pandemic

The modality of routine healthcare provision in the state of Qatar had to be switched from in-person to virtual (phone/video) consultations during the peak of the pandemic to contain the dissemination of the deadly virus [13]. The mental health service at HMC responded quickly to the developing situation. The community mental health services including the outpatient CAMHS service were provided video equipment and software to ensure continuity of care to the patient population.

The online SLP interventions were conducted in real-time audio and/or video connection through high-speed internet to create an experience close to that achieved in an in-person encounter. The parent/caregiver was expected to attend the session to facilitate the intervention. Parents were trained to support the learning of their children during sessions through prior completion of training tasks. The tasks were emailed to parents/caregivers in between sessions to facilitate their preparedness for the session. The SLP activities included direct tasks involving auditory or auditory-visual stimulation. Several resources were used e.g. flashcards, social squad videos, games focusing on the enhancement of several communication skills, and conversational activities, etc. The use of social squad videos combined with checking the child’s understanding of facial expressions, gestures, intonations, etc. proved particularly helpful in engaging children with ADHD, ASD (high functioning), and social communication disorder.

The challenges associated with this sudden transition to virtual modalities of treatment were twofold. Firstly, the impact on patients, particularly those who were at a higher risk of deterioration in their mental state and behaviour due to this sudden change. Secondly, the challenge of speech-language pathologists having to adapt to a new way of delivering therapy since many were using this delivery method for the first time globally [14].

The CAMHS outpatient service at HMC has two speech-language pathologists as part of its MDT (one male and one female). In terms of the clinical activity in the two clinics, a total of 709 appointments (new and follow-up) were offered during the pre-pandemic period, 1601 during the pandemic, and 1668 during the post-pandemic year. The relatively lower number in the pre-pandemic period could be explained by the absence of a dedicated speech-language pathologist between March 2019 and August 2019 due to the previous therapist leaving the service. This gap was bridged to some extent by a visiting therapist who could offer limited input only. To make a more reliable comparison, we also reviewed the average number of sessions per month and the number of time units consumed (each unit comprising 15 min) per month during the three periods. It was noted that the average number of sessions offered increased across the three periods and by 65.3% between the pre-pandemic and post-pandemic periods. The increase in the number of time units (90-minute new-patient and 45-minute follow-up appointments) was even more pronounced.

We also reviewed the utilisation of different contact modalities (Telephone, video, in-person) during the pandemic and post-pandemic periods. We were able to obtain the contact modality record for 1428 appointments out of 1601 for the pandemic period and 1387 out of 1668 for the post-pandemic period. We noted that 58.3% of all appointments during the pandemic were conducted via telephone, followed by video (22.2%) and in-person contact (19.5%). The high number of telephone appointments during this year can be explained by the fact that between March 15 and Jul 1, 2020, in-person contact had to be almost discontinued due to the severity of the pandemic (first wave peaking in May 2020) and until suitable video equipment and privileges were put in place.

Telephone consultations during the first three months mainly involved providing counseling to existing patients during follow-up appointments. Telephone interventions were complemented with the completion of worksheets (emailed soft copies) to make the virtual learning experience more productive. The post-pandemic period, however, saw a major decrease in the proportion of telephone appointments (22%) whereas video and in-person appointments increased to 39.7% and 38.3% respectively. A total of 868 appointments were held via video over the two years which formed 30.8% of all appointments offered during the two years.

### Feedback from families

Previously, high patient satisfaction has been reported with telehealth across ages and conditions for paediatric patients [15]. We were interested to know whether the provision of virtual SLP services by HMC CAMHS to children and young people during the pandemic had impacted the quality of care and service standards. Parents were asked to rate their responses on a typical Likert scale ranging from 1 (strongly disagree) to 5 (strongly agree). Levels 2, 3, and 4 corresponded to “disagree”, “neutral”, and “agree” respectively.

A total of 32 families provided their feedback. 84.4% agreed or strongly agreed when asked whether their expectations from the sessions were met and whether they believed progress was made toward the goals. Interestingly, more than two-thirds of parents found the virtual intervention as effective as an in-person session for their children. Three-quarters of the parents found a virtual consultation more helpful than canceling the appointment altogether, in situations where an in-person consultation could not be carried out (Table 3).

**Table 3:** Feedback from parents

	Strongly agree	Agree	Neutral	Disagree	Strongly disagree
**Q1 My expectations were met**	14 (43.8%)	13 (40.6%)	4 (12.5%)	1 (3.1%)	0
**Q2 Progress was made toward achievement of goals**	12 (37.5%)	15 (46.9%)	3 (9.3%)	2 (6.3%)	0
**Q3 Therapist was accessible and professional**	24 (75%)	6 (18.8%)	1 (3.1%)	1 (3.1%)	0
**Q4 Session proved as effective as an in-person one**	7 (21.9%)	15 (46.9%)	3 (9.3%)	7 (21.9%)	0
**Q5 The session proved a lot more productive than cancellation**	14 (43.8%)	10 (31.3%)	5 (15.6%)	3 (9.3%)	0
**Q6 I would recommend tele-speech therapy to other families**	9 (28.1%)	10 (31.3%)	7 (21.9%)	5 (15.6%)	1 (3.1%)

## Discussion

In the context of advancing epidemic preparedness for health systems, the impact of the COVID-19 pandemic on healthcare services, particularly for children and adolescents, has come to the forefront. This study sheds light on the dynamic response of speech-language pathology (SLP) services within a specialized outpatient child and adolescent mental health service (CAMHS) to the challenges posed by the pandemic. The surge in demand for SLP services, particularly for social communication challenges and autism spectrum disorder, highlights the significance of early intervention in maintaining the developmental trajectory of this population. It was anticipated at the beginning of the pandemic that disruption to SLP services for children could contribute to the persistence of speech and language problems among them. To meet this challenge, the need for SLP clinicians to actively provide telehealth interventions during the pandemic was therefore recommended [16]. 

A considerable amount of literature in recent times has highlighted the potential impact of the pandemic on the social and communication development of children, particularly those with vulnerabilities [17, 18]. The social restrictions during the COVID-19 pandemic adversely impacted the learning disability population significantly [19] as it caused disruption to their access to healthcare services, including SLP interventions. A study in the United Kingdom concluded that many parents observed a worsening of social-communication skills among their children with autism spectrum disorder during COVID-19 [20]. This conclusion is consistent with the notable finding of our study of the number of referrals for children and adolescents with social communication challenges increasing consistently over the 3 years. Furthermore, the number of these specific referrals increased more than 3-fold when the pre and post-pandemic periods were compared. The number of referrals for children with autism spectrum disorder, a condition characterized by social communication deficits and often associated with speech delay, also increased by 60%. The findings of this study highlight the significant impact of the COVID-19 pandemic on Speech-language pathology (SLP) services in a specialized outpatient child and adolescent mental health service (CAMHS). The surge in demand for SLP services for children and adolescents suggests the profound effect of the pandemic on the mental health, speech, and social communication of this special population.

The significant increase in the adoption of virtual modes of contact for speech and language intervention during the pandemic served the purpose of a “window of opportunity” for both healthcare services and families to consider remotely delivered interventions in the longer term. This was demonstrated by the two-fold increase in the utilisation of video contact in HMC CAMHS even after the end of the worst period of the pandemic. The successful adoption of telehealth technology for delivering SLP services emerged as a significant outcome of this study. Telehealth SLP interventions proved to be an effective alternative to in-person sessions during the pandemic. Many families reported satisfaction with the service, as it offered convenience and accessibility and yet remained reasonably effective. Telehealth SLP services may therefore have the potential to be integrated into clinical practice even beyond the pandemic as demonstrated in this study. Incorporating telehealth SLP services into CAMHS can improve mental health provision and address the surge in demand for SLP services. The barriers that may still exist (no access to suitable equipment or broadband and lack of training) globally preventing judicious and effective utilisation of tele/video consultations in SLP services [14, 21] will need addressing as a matter of priority to embrace this much-needed intervention effectively.

### Lessons gained and future directions

The lessons gained from this paper provide valuable insights into the opportunities and challenges presented by the COVID-19 pandemic in the context of speech-language pathology (SLP) services for children and adolescents with neurodevelopmental and mental disorders. This paper highlights the significant impact of the pandemic on the demand for SLP services, particularly among individuals with social communication challenges and autism spectrum disorder. The adoption of telehealth modalities, including phone and video consultations, proved to be an effective and accessible alternative during the pandemic. Feedback from families indicated a positive response to telehealth interventions, with many parents finding them effective and helpful. Importantly, the study underscores the potential of integrating telehealth SLP services into clinical practice beyond the pandemic to meet the increasing demand for mental health interventions among children and adolescents. The findings emphasize the need for long-term evaluation of telehealth services and for addressing potential barriers to access, such as availability of suitable equipment and broadband. The paper’s insights have implications for the future of healthcare delivery, suggesting the importance of sustaining telehealth options, training healthcare professionals in virtual care delivery, and engaging policymakers to ensure equitable access to these services. By highlighting these lessons, the paper contributes to advancing epidemic preparedness for health systems by showcasing innovative practices that can enhance the resilience of healthcare services in the face of challenges like pandemics.

This study provides a timely contribution to the discourse on advancing epidemic preparedness for health systems. The findings of this study suggest several future directions that can be considered to optimize care for children and adolescents receiving SLP interventions. There is a need for long-term evaluation in the form of conducting longitudinal studies to explore the lasting impact of telehealth SLP services on treatment outcomes and service satisfaction. Studies allowing comparison of the effectiveness of telehealth interventions with conventional in-person therapy may identify new horizons for children and adolescents with different neurodevelopmental and behavioral disorders.

There is a need for investigating potential barriers in accessing telehealth SLP services, particularly among low-income families and those living in remote areas with no access to Internet. The training of SLP clinicians in delivering telehealth interventions is crucial and needs to be expanded across services. Furthermore, the engagement of policymakers to ensure sustainable funding for training and securing telehealth equipment will help support the long-term integration of telehealth SLP services into mainstream healthcare systems. The aforementioned suggestions can help CAMHS in responding effectively to the increased global demand of SLP service and continue to provide high-quality care to its patient population as we emerge from the pandemic.

## Conclusion

The pandemic has impacted considerably on the social communication of children who have underlying neurodevelopmental or mental disorders. Telehealth services can be helpful in supporting children with speech and language and social communication challenges in outpatient CAMHS settings. The demand for CAMHS services including the need for SLP interventions has escalated even further since the onset of the pandemic. The successful implementation of telehealth interventions underscores the potential for innovation in service delivery, identifying digital healthcare as a key tool for future epidemic preparedness.

### Electronic supplementary material

Below is the link to the electronic supplementary material.

Supplementary Material 1

## Data Availability

No further data is available other than what is being published.
