# Supplementary material for: Epidemic preparedness and innovations in digital healthcare: enhancing post-pandemic speech-language pathology services for child and adolescent mental health in Qatar
Source: BMC Health Serv Res. 2024 May 28;24:673. doi: 10.1186/s12913-024-10989-y (PMC11134672; doi:10.1186/s12913-024-10989-y)
Supplement: Supplementary file 1 — Supplementary Material 1 [file 12913_2024_10989_MOESM1_ESM.docx]

**FEEDBACK QUESTIONNAIRE FOR SPEECH-LANGUAGE PATHOLOGY SERVICES IN HMC CAMHS**

For each question, respondents can choose one of the given options:

**Question 1: My expectations from the tele-speech intervention were met.**

Strongly Agree

Agree

Neither Agree nor Disagree

Disagree

Strongly Disagree

**Question 2: Progress was made toward the achievement of the speech-language goals through the tele-speech therapy session.**

Strongly Agree

Agree

Neither Agree nor Disagree

Disagree

Strongly Disagree

**Question 3: The speech-language therapist approached the session professionally and was easy to reach.**

Strongly Agree

Agree

Neither Agree nor Disagree

Disagree

Strongly Disagree

**Question 4: The tele-speech therapy session proved as effective as an in-person session for my child.**

Strongly Agree

Agree

Neither Agree nor Disagree

Disagree

Strongly Disagree

**Question 5: Having the tele-speech therapy session instead of an in-person session proved a lot more productive for my child than total cancellation of the appointment due to infection control or other practical reasons.**

Strongly Agree

Agree

Neither Agree nor Disagree

Disagree

Strongly Disagree

**Question 6: I would recommend tele-speech therapy to other parents and families.**

Strongly Agree

Agree

Neither Agree nor Disagree

Disagree

Strongly Disagree
